# Health risks from consumption of medicinal plant dietary supplements

**DOI:** 10.1002/fsn3.1636

**Published:** 2020-05-19

**Authors:** Małgorzata Ćwieląg‐Drabek, Agata Piekut, Iwona Szymala, Klaudia Oleksiuk, Mehdi Razzaghi, Weronika Osmala, Konstancja Jabłońska, Grzegorz Dziubanek

**Affiliations:** ^1^ Department of Environmental Health Faculty of Health Sciences in Bytom Medical University of Silesia Katowice Poland; ^2^ Provincial Sanitary and Epidemiological Station Katowice Poland; ^3^ Department of Epidemiology Faculty of Health Sciences in Bytom Medical University of Silesia Katowice Poland; ^4^ Department of Mathematical and Digital Sciences Bloomsburg University Bloomsburg PA USA

**Keywords:** cadmium, dietary supplements, health risk assessment, heavy metals, lead, medicinal plants, mercury

## Abstract

The aim of this study was to determine the heavy metal contents of dietary supplements manufactured from medicinal plants and assess the potential daily burden on their consumers. The study consisted of 41 dietary supplements produced from terrestrial plants or microalgae. The analysis of cadmium, lead, and mercury content was performed using analytical methods. The content of Cd and Pb was determined by flame atomic absorption spectrometry (FAAS). The mercury content was determined using atomic absorption spectrometry with the generation of cold mercury vapor (CVAAS). The presence of at least one of the three analyzed heavy metals was found in 79.2% samples of supplements produced from terrestrial plants and in 88.2% supplement samples produced from microalgae. Hazard quotient was used to calculate noncarcinogenic risk for humans by ingestion of dietary supplements containing heavy metals. From among all supplements, 68.3% of samples were contaminated with Cd and Pb (this does not always apply to the same samples) and 29.3% of samples were contaminated with Hg. The health risk assessment of consumers of dietary supplements showed, in an extreme case, that taking this supplement for only one week poses a health risk associated with exposure to Pb. The health risk associated with the intake of dietary supplements primarily depends on the duration of consumption.

## Graphical abstract

The aim of this study was to determine the heavy metal contents of dietary supplements manufactured from medicinal plants and assess the potential daily burden on their consumers. The study consisted of 41 dietary supplements produced from terrestrial plants or microalgae. The analysis of Cd, Pb, and Hg content was performed using analytical methods. Hazard quotient was used to calculate noncarcinogenic risk for humans by ingestion of dietary supplements containing heavy metals.

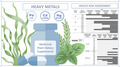

## 1 INTRODUCTION

Dietary supplements, defined as foodstuffs, are readily available to the public, and their marketing campaigns are held on a broad scale. Interest in such preparations is surging, and the food supplement market is being developed with overwhelming and unprecedented dynamics. Dietary supplements are products increasingly chosen as an easy way to enrich the daily diet with vitamins and minerals. There are classified as special category of foods and are subject to food law, not pharmaceutical regulations. As a result, the requirements to dietary supplements are much less restrictive than to medications. According to EFSA, 2019; EP, 2002, dietary supplements are “foodstuffs whose purpose is to complement a regular diet. They are a concentrated source of vitamins, minerals, or other substances (both simple and complex) ensuring a nutrition or other physiological effect. They occur in a form making dosing possible” (EFSA, 2019). Both the form of the supplement (tablets, capsules, drops, liquid, or powder) and their most common place of distribution, that is, pharmacies, may mislead the consumer and suggest their association with medicine.

In many European countries, including Poland, in case of dietary supplements, there is no need for statutory requirement regarding continuing supervision of their security (EP, 2002). The idea of taking food supplements is not to cure diseases. The goal is to lower the consequences of risk factors influencing the consumer on everyday basis and may cause the disease development. Since those supplements do not cure, it is hard to predict the side effects of their usage.

Currently, society's knowledge of dietary supplements is primarily channeled through the marketing displays of products. With the dynamically evolving markets for dietary supplements, especially in the last decade, public awareness of their possible harm is very slim. In 2015, dietary supplement market was worth € 0.8 billion in Poland. According to Industry Report, in the next 5 years the market will be progressing by 8% per year, and by the year 2020, it will have exceeded €1.2 billion (IR, 2017).

Until recently, mostly health‐promoting components like vitamins, minerals, or amino acids could be found in dietary supplements. Currently, they can also contain raw materials of plant and herbal origin or their extracts (Filipiak‐Szok, Kurzawa, & Szłyk, 2015). Food supplements on the base of plants, not only available in pharmacies, but also herbal stores or via Internet retailers may be contaminated with toxic elements. Thus, they could become an additional source of absorption of such elements by the human body. Significant amounts of heavy metals, especially Cd and Pb in some of the herbaceous plants, can propagate from their growth in high‐intensive traffic or industrialized areas (Socha & Borawska, 2011). In some special circumstances, the use of supplements may be necessary and advisable. However, over‐usage, leisurely consumption, or exposure to higher than the recommended allowable intake can cause toxicity and pose serious health threats (Korfali, Hawi, & Mroueh, 2013).

Despite the worldwide use of botanical products and increase in popularity, the WHO found that among the 191 listed countries, only 25 had a national policy regarding herbals and only 64 regulated them (Avigan, Mozersky, & Seeff, 2016). Polish dietary supplement market responsible for nutrition safety is known to be improperly monitored. According to the State Control (Kotynia, Szewczyk, & Tuzikiewicz‐Gnitecka, 2017), Poland does not offer the right security of dietary supplements to its citizens and quality of products was not effective either.

The primary aim of this study was to determine the Cd, Pb, and Hg contents in dietary supplements based on the type of plants (terrestrial or microalgae) from which they are produced. Our secondary goal was to assess the potential daily heavy metal burden on their consumers.

## 2 MATERIALS AND METHODS

### 2.1 Product Selection and Sampling

The study material consisted of 41 dietary supplements, available on the Polish market, produced from terrestrial plants (24 samples) or from microalgae (17 samples). The characteristics of all samples are summarized in Table 1. Samples were collected in accordance with the methods set out in Commission Regulation (EC) No 333/2007 of 28 March 2007 dictating methods for the sampling and analysis for the control of levels of trace elements and technology process impurities in foodstuffs (EC, 2007). Samples for testing were collected under the official control of the trade, in a manner that would ensure the representativeness of the lot. To determine the content of lead and cadmium, the size of the single sample was 5–15 g, depending on the type of product. The size of an individual sample to determine the content of mercury was 1–7 g depending on the type of product.

**Table 1:** Characteristics and concentration of heavy metals (Cd, Pb, Hg) with dietary supplements produced from terrestrial plants (TP) and from microalgae (M)

Sample ID	Origin	Main ingredients	Form of supplement	Pb [mg/kg]	LOD_Pb_ [mg/kg]	Cd [mg/kg]	LOD_Cd_ [mg/kg]	Hg [mg/kg]	LOD_Hg_ [mg/kg]
TP1	UE	Cornelian, dandelion	tea bag	<LOD	0.020	0.020	0.005	<LOD	0.0040
TP2	Poland	Sage	tablets	<LOD	0.020	<LOD	0.005	<LOD	0.0040
TP3	Poland	Flax	tablets	<LOD	0.020	<LOD	0.005	<LOD	0.0040
TP4	Poland	Extract of peppermint, fennel and cumin	tea bag	<LOD	0.020	0.075	0.005	<LOD	0.0040
TP5	Poland	Artichoke herb, primrose	tea bag	0.191	0.020	0.080	0.005	<LOD	0.0040
TP6	Poland	Nettle	tea bag	0.054	0.020	<LOD	0.005	<LOD	0.0040
TP7	Poland	Nettle	dried herbals	0.067	0.020	<LOD	0.005	<LOD	0.0040
TP8	Poland	Shells of the seed plantain Ispaghula	powder	<LOD	0.020	0.011	0.005	<LOD	0.0040
TP9	Switzerland	Extract of sage, vitamin C	tablets	<LOD	0.020	<LOD	0.005	<LOD	0.0040
TP10	Switzerland	Extract of thyme and plantain	tablets	<LOD	0.020	<LOD	0.005	<LOD	0.0040
TP11	Poland	Xylitol	powder	<LOD	0.020	0.009	0.005	<LOD	0.0040
TP12	Poland	Blackcurrant leaves, hibiscus, chokeberry fruits	dried herbals	0.021	0.020	<LOD	0.005	<LOD	0.0040
TP13	Poland	Melissa extract	tablets	<LOD	0.020	<LOD	0.005	<LOD	0.0040
TP14	Poland	Cistus	tea bag	0.460	0.005	0.130	0.001	0.013	0.0006
TP15	Poland	Cistus	dried herbals	0.210	0.003	0.940	0.001	‐	‐
TP16	Poland	Cistus	dried herbals	0.110	0.003	0.004	0.001	0.009	0.0040
TP17	Albania	Smallflower hairy willowherb	tea bag	0.140	0.005	0.045	0.001	0.008	0.0006
TP18	Poland	White mulberry	tablets	0.047	0.003	0.011	0.001	<LOD	0.0040
TP19	China	Radix rehmanniae preparata, fructus corni officinalis	tablets	0.690	0.015	0.031	0.003	0.006	0.0008
TP20	China	Radix rehmannia glutinosa, wolfiporia	tablets	3.700	0.015	0.160	0.003	0.028	0.0008
TP21	China	Arisaema cum bile, radix aconiti carmichaeli preparata	tablets	0.290	0.015	0.036	0.003	0.006	0.0008
TP22	China	Radix bupleuri chinensis, radix angelicae sinensis	powder	1.100	0.015	0.099	0.003	0.025	0.0008
TP23	China	Rhizoma cibotii, radix angelicae pubescentis	tablets	2.600	0.015	0.170	0.003	0.013	0.0008
TP24	China	Radix rehmanniae preparata, sclerotium poriae cocos	tablets	0.370	0.015	0.038	0.003	0.008	0.0008
M1	Poland	Spirulina	tablets	<LOD	0.020	<LOD	0.005	<LOD	0.0040
M2	USA	Spirulina	tablets	<LOD	0.020	0.037	0.005	‐	‐
M3	Poland	Spirulina	tablets	0.316	0.020	0.012	0.005	<LOD	0.0040
M4	Poland	Spirulina	tablets	0.340	0.020	<LOD	0.005	<LOD	0.0040
M5	Poland	Spirulina	tablets	0.240	0.020	0.056	0.005	<LOD	0.0040
M6	Poland	Spirulina	powder	0.270	0.020	0.078	0.005	<LOD	0.0040
M7	Poland	Spirulina	powder	0.064	0.020	0.063	0.005	<LOD	0.0040
M8	Poland	Spirulina	tablets	0.180	0.020	0.012	0.005	<LOD	0.0040
M9	Poland	Spirulina	powder	0.079	0.005	0.024	0.001	0.008	0.0008
M10	Poland	Spirulina	powder	0.076	0.003	0.022	0.001	‐	‐
M11	USA	Spirulina	tablets	0.066	0.003	0.019	0.001	<LOD	0.0040
M12	China	Spirulina	powder	0.067	0.003	0.041	0.001	‐	‐
M13	Poland	Spirulina	tablets	0.380	0.005	0.062	0.001	0.016	0.0008
M14	Poland	Chlorella	tablets	0.089	0.020	<LOD	0.005	<LOD	0.0040
M15	Poland	Chlorella	tablets	0.052	0.020	<LOD	0.005	<LOD	0.0040
M16	Poland	Chlorella	powder	<LOD	0.020	0.036	0.005	0.017	0.0040
M17	Taiwan	Chlorella	tablets	<LOD	0.020	<LOD	0.005	<LOD	0.0040
**MPC** ^a^				**3.0**		**3.0 for M**		**0.1**	
						**1.0 for TP**			

^a^ MPC—maximum permissible concentration of heavy metals in dietary supplements [mg/kg] according to (EC) 629/2008 (EC, 2008).

The samples were collected by District Sanitary and Epidemiological Stations, mainly from the market, including pharmacies, on the basis of annual sampling plans for food testing under official food control and monitoring for the State Sanitary Inspection, prepared by the Chief Sanitary Inspectorate. These plans in the case of testing dietary supplements include the following:—directions of tests specified in the Commission Regulation (EC) No. 1881/2006 of 19 December 2006 specifying maximum levels of certain contaminants in foodstuffs (EC, 2006):

—analysis of applications under the European and national Rapid Alert System for Food and Feed (RASFF) in recent years,

—data from monitoring and official control in the field of metal pollution from previous years,

—analysis of the structure of consumption of supplements,

—population and market share of domestic and import samples.

The implementation and analysis of all the above‐mentioned factors in creating a sampling plan for research are to ensure the representativeness of the results.

All sample material delivered to the laboratory was used to prepare the laboratory sample. As far as possible, during sample preparation, the apparatus and equipment in contact with the sample did not contain metals and were made of an inert material, for example, plastic (polypropylene—PP, polytetrafluoroethylene—PTFE) or glass, and were washed with acid to minimize the risk of contamination. In the sample preparation, the procedures described in the EN 13,804:2013 standard were used (CEN, 2013).

### 2.2 Analytical methods

The analysis of Cd, Pb, and Hg content was performed at the accredited Integrated Laboratory of the Provincial Sanitary and Epidemiological Station in Katowice, Poland, using analytical methods that meet the criteria specified in Commission Regulation (EC) No. 333/2007 of 28 March 2007 (EC, 2007) for methods applicable for the official control of food. The method of determination of lead and cadmium content was based on the dry mineralization of samples and on the determination of the content of these metals by flame atomic absorption spectrometry (FAAS) after extraction with methyl isobutyl ketone (MBIK) of the metal complex with ammonium 1‐pyrrolidinedithiocarbamate (APDC). Samples for testing before analysis were homogenized. Depending on the type of sample, 5–15 g of the sample was weighed into quartz evaporators. The samples were charred and then incinerated in a muffle furnace at a temperature not exceeding 450°C. The resulting ash was dissolved in 1 N nitric acid, and then, the extraction of lead and cadmium with methyl isobutyl ketone was performed after the metal was first bound with the ammonium 1‐pyrrolidinedithiocarbamate complex. The content of metals was determined by flame atomic absorption spectrometry using a wavelength of 283,3 nm for lead and 228,8 nm for cadmium. The mercury determination method consisted of wet mineralization of samples in a mixture of acids in the presence of a catalyst and determination by atomic absorption spectrometry with the generation of cold mercury vapor (CVAAS). Samples for testing before analysis were homogenized. Depending on the type of sample being tested, 1 to 7 g of the sample was weighed. In the process of sample wet mineralization, mixture of nitric and sulfuric acid and a catalyst in the form of vanadium pentoxide were used. The mineralized samples were quantitatively transferred to 100‐ml graduated flasks and the mercury content determined therein after the reduction of inorganic mercury compounds (Hg^2+^) to metallic mercury (Hg^0^) by atomic absorption spectrometry with the generation of cold mercury vapor. All samples were tested in two parallel determinations. The final result of the determination was the arithmetic mean of two results differing not more than by 15% of the lower result. Blind reagent samples were prepared in parallel with samples tested under the same conditions without the addition of a matrix to take into account contaminants from the solutions and reagents used.

### 2.3 Human health risk assessment

The daily dietary intake of Cd, Pb, and Hg by consumers of dietary supplements was calculated for health risk assessment based on recommendations of producers of particular preparations specifying the daily dose that should be consumed to obtain the intended effect. According to the Commission Regulation No 420/2011 and No 488/2014 setting maximum levels for certain contaminants in foodstuffs, the maximum permissible concentration (MPC) of analyzed metals in the food was used for all the scenarios (EC, 2011, 2014).

The average daily dose (ADD) of ingested Cd, Pb, and Hg by adult consumers of analyzed dietary supplements, depending on scenario, was calculated using the following equation recommended by the US EPA (US EPA, 2011):

ADD=C×IR×EF×ED/BW×AT

where.

‐ ADD is the average daily potential dose of heavy metal through ingestion (mg/kg day^‐1^);
‐ C is the metal content in the food product (mg/kg);
‐ IR is the ingestion rate (kg/day);
‐ EF represents the exposure frequency (365 days^‐1);^
‐ ED is the exposure duration (70 years);
‐ BW is the body weight (70 kg);
‐ AT represents the average exposure time (EF x ED).

Hazard quotient defined by the following equation:

HQ=ADD/RfD

was used to calculate noncarcinogenic risk for humans as the result of ingestion of dietary supplements containing heavy metals. According to the Environmental Protection Agency (EPA) document (US EPA, 2011), the hazard quotient less than 1 is assumed to be safe, and HQ values equal to or exSceeding 1 may pose potential noncarcinogenic effects. Reference dose (RfD) is the tolerable daily intake of the contaminant (mg/kg day^‐1^) via the oral exposure. The reference doses for Cd were 1 mg/kg (for terrestrial plants) and 3 mg/kg (for microalgae) (EC, 2014). The reference doses for Pb and Hg were 3.0 mg/kg and 0.1 mg/kg, respectively (EC, 2011).

For the risk assessment, two scenario groups were considered: one based on dosage level and the other based on longevity and period of intake of the supplements. The first group was divided into three cases of MPC (scenario I), recommended maximum daily dose stated on the package (scenario II) and twice the recommended daily dose (scenario III). For each case, the average daily dietary exposure (ADD) and the hazard quotient (HQ) were calculated (Tables S5, S7, and S9, in Appendixes). In the second scenario group, it was assumed that the consumer was taking the maximum recommended dose stated on the package, but the duration of usage was allowed to vary. In this scenario group, we considered four cases of two weeks (scenario IV), one month (scenario V), two months (scenario VI), and three months (scenario VII) exposure, respectively (Tables S6, S8, and S10, in Appendixes). For the purpose of risk assessment, exposure period of two weeks was assumed to be the shortest since that is normally the period that one package of the supplement lasts, although there is evidence to show that in general, consumers use supplements for longer periods of time. Kozlowski et al. (SAO, 2016) give the average time for consumption of the dietary supplements in Poland as two to three months.

To estimated chronic noncancer hazards for multiple heavy metals via dietary intake, hazard index (HI), recommended by the US EPA, was used (US EPA, 1989, 2011). Hazard index is based on the sum of HQs calculated for individual heavy metals that cause similar adverse health effects. The following equation estimates the HI from exposure to multiple elements:

$$\text{HI}={\text{HQ}}_{1}+{\text{HQ}}_{2}+\;\ldots \;+{\text{HQ}}_{i}$$

where.

‐ HI is the hazard index for chronic exposure to heavy metals 1 through *i*, unitless
‐ HQ*_i_* is the hazard quotient for the *i*th heavy metal, where all *i* heavy metals are assumed to affect the same target organ or organ system, unitless.

As with the HQ, an HI value less than or equal to 1 indicates that the exposure is not likely to result in adverse noncancer effects. According to US EPA, an HI value greater than 1, however, does not necessarily suggest a likelihood of adverse health effects and cannot be interpreted as a statistical probability of adverse effects occurring.

## 3 RESULTS AND DISCUSSION

### 3.1 Concentrations of Cd, Pb, and Hg in Dietary Supplements

In the current study, samples were coded according to whether they were produced from terrestrial plants (TP) or from microalgae (M). From the 41 analyzed supplements, 68.3% of samples (*n* = 28) were contaminated with Pb and Cd (this does not always apply to the same samples) and 29.3% of samples (*n* = 12) were contaminated with Hg. The presence of at least one of the three analyzed heavy metals was found in 79.2% samples of supplements produced from terrestrial plants (*n* = 19) and in 88.2% supplement samples produced from microalgae (*n* = 15).

In Polish legislation, there is no separate category for permissible heavy metal content in dietary supplements. The assessment of dietary supplements of heavy metals is carried out on the basis of the Commission Regulation (EC) No 629/2008 of 2 July 2008 amending Regulation (EC) No 1881/2006 setting maximum levels for certain contaminants in foodstuffs (EC, 2008), in which the MPC of contaminants, including Cd, Pb, and Hg, was determined. The concentration of Pb in one sample (TP20) exceeded the MPC of 3.0 mg/kg, and in another sample (TP23), it was 2.7 mg/kg, almost equivalent to the MPC. Both supplements are produced in China, and their main components are terrestrial plants. The highest concentration of Cd and Hg (0.94 mg Cd/kg and 0.03 mg Hg/kg) was determined in two terrestrial plant supplements, respectively, in TP15 and TP20 (Table 1).

Table 2 shows descriptive statistics for concentrations of heavy metals in dietary supplements depending on the main ingredient from which were produced. The concentrations of Cd, Pb, and Hg in the analyzed supplements produced from terrestrial plants ranged from < Limit of Detection (< LOD) – 0.9 mg/kg, from < LOD – 3.7 mg/kg, and from < LOD – 0.03 mg/kg, respectively. In case of supplements produced from microalgae, the minimum and maximum values of analyzed heavy metals amounted: < LOD – 0.4 mg/kg for Pb, < LOD – 0.08 mg/kg for Cd, and < LOD – 0.02 mg/kg for Hg (Table 2).

### 3.2 Health Risk Assessment

The associated risk presented in this study was based on two kinds of scenarios of exposure from dietary supplements (Tables S5 – S10, in Appendixes). The first group included three scenarios in which ADD and HQ were estimated depending on the adopted dose of individual supplements by consumers. The risk assessment based on the first three scenarios was measured given the consumption of a dietary supplement during one day, but in the first scenario as the concentrations of Cd, Pb, and Hg, the MPCs for these elements were given (EC, 2006, 2008, 2011, 2014). The second and third scenarios took into account the actual contents of the analyzed heavy metals in all supplements. However, the differentiating factor was the maximum dose of supplement recommended by the producer to achieve the desired health effect (scenario II) and twice the maximum dose (scenario III) (Tables S5, S7, and S9, in Appendixes). In the second group, there were four scenarios in which ADD and HQ were estimated depending on the period of consumption of dietary supplements (scenarios IV‐VII) (Tables S6, S8, and S10, in Appendixes). The period of consuming the dietary supplement was analyzed, from two weeks (for most supplements, two weeks is the period during which one package is used) to two and three months (it is the average time of consuming dietary supplements in Poland (SAO, 2016)). In each scenario, the highest daily average dose (ADD) was estimated for all three metals, taking into account the maximum dose of analyzed supplements recommended by the producers. The HQ for Cd and Pb for each dietary supplement in the three scenarios, and for mercury in scenarios I to IV, were found below 1, which indicates safe with no risk to human health. Higher than 1 HQ value for Cd was estimated for dietary supplement from Poland no. TP15 (cistus) in scenarios IV‐VII (HQ was from 1.9 to 11.3), and in two Chinese products—in sample no. TP20 in VI and VII scenarios (HQ was from 1.1 to 1.6) and TP22 in scenario VII (HQ = 1.4). It means that consuming this dietary supplement in the maximum recommended dose for just two weeks for TP15, 2 months for TP20, and 3 months for TP22 may pose a noncarcinogenic risk of consumers health (Table 3). The assessment of the health risk to consumers of dietary supplements related to Pb contents showed a significant risk for four Chinese supplements: TP20, TP22, TP23, and TP24 and for sample M13 (spirulina, from Poland) with an intake period of at least 2 weeks for TP20 (HQ was from 2.1 to 12.6), 4 weeks for TP22 (HQ was from 1.8 to 5.3), 4 weeks in case of TP23 (HQ was from 1.2 to 3.7), 2 months in case of TP24 (HQ was from 1 to 1.5), and 3 months in case of M13 (HQ = 1.1) (Table 3). Estimation of exposure to mercury has shown HQ over 1 in 5 samples, for example, in TP17 from Albania (HQ = 1.1), TP20 (HQ = 1.9–2.9), TP22 (HQ = 1.2–3.6), and two samples from Poland—M9 (HQ = 1) and M13 (HQ = 1.5) (Table 3). HQ values higher than 1 are bolded in the tables placed in Appendixes.

**Table 2:** Statistical summary of the content of heavy metals (Cd, Pb, Hg) [mg/kg] in dietary supplements produced from terrestrial plants (TP) and microalgae (M)

Heavy Metal	SAMPLE ID									
	TP1 ‐ TP24					M1‐M17				
	AM	*SD*	ME	MIN	MAX	AM	*SD*	ME	MIN	MAX
Pb	0.419	0.896	0.061	<LOD	3.700	0.131	0.130	0.076	<LOD	0.380
Cd	0.077	0.191	0.016	<LOD	0.940	0.027	0.026	0.022	<LOD	0.078
Hg	0.005	0.008	<LOD	<LOD	0.028	0.003	0.006	<LOD	<LOD	0.017

Abbreviations: AM, arithmetic mean; MAX, highest concentration; ME, median; MIN, lowest concentration; *SD*, standard deviation.

Hazard index (HI), based on the sum of HQs calculated for individual heavy metals, was calculated in dietary supplements where there were two or all three of analyzed metals (Cd, Pb, and Hg) (Table S11, in Appendixes). The cumulative HI for cadmium and lead was calculated in 11 samples. In the same number of dietary supplement samples, HI for cadmium, lead, and mercury was calculated. HI for cadmium and mercury was calculated in one sample. In the first group of scenarios, no increase in noncancer health risks was demonstrated. In the second group, in some cases HI values exceeded 1 (Table 4). HI for TP15, in case of cumulative exposure to Cd and Pb, was in the range from 2.020 to 11.504. Considering all metals, HI over 1 was recorded in scenarios IV‐VII in the samples TP20 (2.845–17.068) and TP22 (1.718–10.306). In sample TP23, HI ≥ 1 was calculated in scenarios V‐VII (1.667–4.998).

**Table 3:** Hazard quotient (HQ) related to exposure to Cd, Pb, and Hg depending on the period of dietary supplement intake. White cell means HQ <1, dark gray HQ≥1

Heavy metal	Dietary supplement	HQ			
		Scenario IV	Scenario V	Scenario VI	Scenario VII
		2‐week intake	1‐month intake	2‐month intake	3‐month intake
Cd	TP15				
	TP20				
	TP22				
Pb	TP20				
	TP21				
	TP22				
	TP23				
	TP24				
	M11				
Hg	TP17				
	TP20				
	TP22				
	M9				
	M13				

Dietary supplements are commonly available with the intention of providing basic balanced diet. Recent large‐scale advertising campaigns about these products have resulted in increased consumer interest in their usage, which means that the dietary supplement market is developing very dynamically. According to the latest report of the Supreme Audit Office, only in Poland in 2015, the number of purchased dietary supplement packages amounted to over 94 million, and estimates indicate that by 2020 this number will increase by about 8% each year (Korfali et al., 2013). Despite the many advantages of dietary supplements, due to the limited control of their composition before they are released for sale (e.g., content of heavy metals), there are doubts as to whether the introduction of these products on a large scale is safe for consumer health.

In Poland, it is the Chief Sanitary Inspector and subordinate offices who control and give license for the sale of dietary supplements. In the case of food supplement, there is no requirement for production of any clinical trials or other kinds of quantitative research. The only requirement is a notification, and it suffices to inform the Chief Sanitary Inspector. Once the notification is submitted, the product can enter the market. It is to be noted that for the case of medicinal products, it is the duty of the Chief Pharmaceutical Inspector and the Office for Registration of Medicinal Products to control the safety of what is permitted to enter the market and also to constantly monitor the quality of the product for safe usage by medics, pharmacists, and other users.

A thorough investigation by Kotynia et al. (2017) reveals that the consumption of dietary supplements has become a modern element of a healthy lifestyle, often a substitute for a balanced diet, compensation for lack of physical activity, or a nostrum for the unfair battle with stress. Among vegetal dietary supplements available on the Polish market, the most remarkable are the extracts made of ginseng, roseroot, cranberry, chokeberry, aloe vera, horsetail, and grapeseeds. Another group of supplements worth mentioning is oils made of primrose seeds, flax, pumpkin seeds, and sea buckthorn (Czajkowska, 2006). Although there are many advantages of diet of plant origin, there appear to be some doubts among researchers as to whether frequent usage of such substances is safe for consumers. Those questions surface due to lack of information of a proper quality regarding long‐term effects on health, ergo allergic reactions, or toxic effects on internal organs (Schlegel‐Zawadzka & Barteczko, 2009).

In the United States, regulatory scientists have become more and more interested in how the risk of hepatotoxicity is connected to the common usage of some of the herbal products. Based on current US laws, all dietary supplements sold domestically are required to be regulated by the Food and Drug Administration (FDA) as a special category of foods. Under this designation, regulatory scientists do not routinely evaluate the efficacy of these products, even though the content variability and phytochemical complexity that often characterize them can be influential. However, it cannot be denied that a progress has been made in the development of methods to qualitatively and quantitatively measure and screen for contaminants and adulterants in such products when hepatotoxicity is recognized (Avigan et al., 2016).

In terms of dietary supplements, from the point of view of public health, the problem is their potential pollution, including heavy metals. Cadmium (Cd), being one of the most dangerous environmental pollutants, is extremely hazardous due to the fast absorption of it by living organisms and the tendency to accumulate in plant and animal tissues (Kaczyńska, Zajączkowski, & Grzybiak, 2015). The transfer of Cd, adsorbed by plants, may be caused by leaking sewage sludge to agricultural soil. That may play a significant role in food chain, and then accumulate in various human organs (Rahimzadeh, Rahimzadeh, Kazemi, & Moghadamnia, 2017). Cd is mostly being absorbed through the root system, but also through the leaves. Collection of that metal is happening through a competition with other ions (e.g., potassium, calcium, magnesium) for the place in membrane transporters. Therefore, Cd is carried easily and a permanent and systematic binding of metal in the human cell can occur (Gambuś & Rak, 2000). If Cd accumulates in a human body and in the food chain, it can lead to acute and chronic intoxications. Noticeable effects on health are diarrhea, stomach pains, bone fracture, reproductive failure, and even possibly infertility, in addition to damage to the central nervous system, immune system, and psychological disorders. The transformation of normal epithelial cells into carcinogenic cells due to inhibiting the biosynthesis of protein is also caused by Cd (Gambuś & Rak, 2000). The most common effects in humans and animals include damage to the liver, lung, kidneys, and causing anemia, osteoporosis, and a wide range of tumors (Kaczyńska et al., 2015). Toxic influence of lead (Pb) on the human body often occurs as hematopoietic system disorders, manifesting itself via inhibition of hemoglobin synthesis, shortening the lifespan of RBCs, and stimulation of erythropoiesis. An excessive absorption of Pb in the body can have a negative influence on Nervous System and as a result of chronic risk—damage of the kidneys and liver (Flora, Gupta, & Tiwari, 2012; Seńczuk, 2005). Pb may also affect pregnancy—it crosses the placenta and has been confirmed to be the reason for intrauterine death, prematurity, and low birthweight (Papanikolaou, Hatzidaki, Belivanis, Tzanakakis, & Tsatsakis, 2005). Mercury (Hg) and most of its compounds are toxic and became environmental pollutants. Protracted poisoning may lead to disorders of the nervous system and damage to the kidneys. In some of the dietary supplements, significant level of quicksilver was detected and reported via Rapid Alert System for Food and Feed (RASFF) (EC, 2008;Socha, Michalska‐Mosiej, Lipka‐Chudzik, & Borawska, 2013). Above all, defense of public health is what should be a primary concern to health officials and levels of pollutants capable of inducing any kind of public health threads in foods and dietary supplements should not surpass the allowable intake levels. The limits of impurities (Cd, Pb, Hg) should be safe and on the lowest possible level to achieve proper benefits with proper usage (EC, 2008). The highest acceptable quantity of heavy metals in dietary supplements, in accordance with Commission Regulation (EC) of 2 July 2008, is presented in Table 1.

There is a wide range of research papers that refer to threats of excessive or unjustifiable use of dietary supplements, and possible interactions between pharmacological preparations and supplements. The number of papers on measurements of heavy metals in supplements is narrow, and research on the hazard connected with supplementation of botanical substances contaminated with heavy metals is infrequent. Research conducted by Socha and Borawska (2011) includes evaluation of the concentration of Cd in botanical raw materials, herbal mixtures, and infusion. In some of the herbal substance specimen, Cd content was above the standards. However, the results of a large number of papers show the fact that there was no violation of norms, although they point out that the level of Pb in dietary supplements sold in pharmacies was higher than the nonpharmacy sales. The maximal level of Pb turned out to appear in botanical supplements with calming effect, sold in pharmacies (Socha, 2010). Currently, the market for herbals and dietary supplements in the United States is estimated to have a value of $62 billion, and the World Health Organization (WHO) expects it to increase to $5 trillion by 2050 (Ajazuddin, 2012).

The number of published research on the content of heavy metals in dietary supplements is not very high. Comparing the results obtained with available literature, all are at similar levels. *Socha et al.* () studied Cd, Pb, and Hg concentrations in dietary supplements available on the Polish pharmaceutical markets and found that none of the analyzed samples exceeded the MPC specified for these metals. The range of Cd, Pb, and Hg content was, respectively, 0.001–0.167 mg Cd/kg, 0.0004–1.752 mg Pb/kg, and 0.0001–0.048 mg Hg/kg. Korfali et al. (2013) also examined Cd, Pb, and Hg in dietary supplements. These metals were detected in all samples, but were at a minimum level and did not exceed MPC. In case of Cd, in eight samples the concentrations were higher than MPC. The ranges of Cd, Pb, and Hg and in all samples were, respectively, 0.08 to 2.9 mg Cd/kg, 0.01–0.41 mg Pb/kg, and 0.01–0.55 mg Hg/kg.

Human Health Risk was assessed using hazard quotient (HQ) and hazard index (HI). Many researchers have used HQ as a reliable measure for evaluation of risk associated with the exposure to heavy metals by digestive route (Chary, Kamala, & Raj, 2008; Jolly, Islam, & Akbar, 2013; Rattan, Datta, Chhonkar, Suribabu, & Singh, 2005). Therefore, here we adopt the same measure to assess the health risk associated with exposure to heavy metals contained in dietary supplements. Although the HQ‐based risk assessment method does not provide a quantitative estimation for the probability of an exposed population experiencing a negative health effect, it factually provides an indication of health risk level due to exposure to pollutants such as heavy metals (Jolly et al., 2013; Jamal et al., 2013). Additionally, hazard index based on the sum of HQs calculated for individual heavy metals was presented in the study. It gave the opportunity to estimate the health risk for simultaneous exposure to three marked heavy metals.

Our study has revealed the presence of Cd, Pb, or Hg in over 90% of the 41 analyzed samples of dietary supplements. It should be emphasized that the products involved in the analysis did not include all available dietary supplements within a given group. For example, the analyzed microalga group included two products—Chlorella and Spirulina. The next research could be extended to include further products, including Dunaliella, Scenedesmus, Aphanizomenon flos‐aquae. Also a group of terrestrial plants could be expanded to include new plant species.

Heavy metals have the ability to accumulate in the body (Jaishankar, Tseten, Anbalagan, Blessy, & Krishnamurthy, 2014), which can play a significant role when consuming dietary supplements for a longer period. The health risk assessment of consumers of dietary supplements carried out in this study showed, in an extreme case (TP15, TP20, TP 22) that taking these supplements for only two week poses a health risk. However, it should be noted that on the packaging of all the analyzed supplements, there is no warning information of the maximum duration of their usage. Therefore, overdosing of this type of products is quite common since there is a belief that herbal products and supplements do not pose any health threats. In addition, combination of different drugs and supplements poses a risk not only in terms of interactions between chemicals in different preparations but also because of heavy metals entering the body from other sources. Consumers should pay more attention to the consumption of dietary supplements and be aware of the negative effects they can cause. To enhance the safety of dietary supplement consumers, it seems necessary to implement a stricter quality control and determine safe dosing regimens. In addition, authorities allowing supplements for human consumption risk should implement assessment analysis before the introduction of the supplement on the market. Analysis of the results also showed that the amount of heavy metal concentrations in the tested dietary supplements may depend on the place of origin of the product. However, the form of the supplement (powder, tea bags, tablets) and the type of the main ingredient (terrestrial plants, microalgae) have less influence on the differentiation of heavy metal content in the product (Table 1).

There is a need for a legislative action to stabilize the current situation. For example, a system could be introduced warning the user that the product has not been tested by the authorities. Also, there could be a regulating procedure to withdraw a flawed supplement from the market or suspend the distribution of doubtful products. Due to the soaring number of plant‐based supplements on the market, their detailed analysis for public health security and possible toxicity is necessary.

## 4 CONCLUSIONS

1. Dietary supplements show contamination with heavy metals; cadmium was found in 68% of all analyzed samples, lead in 68%, and mercury in 29%.
2. The concentration of heavy metals depends on the country where the dietary supplement was produced. Less important seemed to be form (powder, tea bags, tablets) and whether or not the base ingredient was microalgae or terrestrial plant.
3. The health risk associated with the intake of dietary supplements primarily depends on the duration of consumption.
4. It is suggested that pharmaceutical companies should include information on the recommended duration of consumption, determined from risk assessment analysis.

## 5 ETHICAL GUIDELINES

Ethics approval was not required for this research.

## CONFLICT OF INTEREST

The authors declare that the research was conducted in the absence of any commercial or financial relationships that could be construed as a potential conflict of interest.

6

**Table 4:** Hazard index (HI) for chronic exposure to heavy metals depending on the period of dietary supplement intake. White cell means HQ <1, dark gray HQ≥1

Cumulative effect of heavy metal exposure	Dietary supplement	HI			
		Scenario IV	Scenario V	Scenario VI	Scenario VII
		2‐week intake	1‐month intake	2‐month intake	3‐month intake
Cd + Pb	TP5				
	TP15				
	TP18				
	M3				
	M5				
	M6				
	M7				
	M8				
	M10				
	M11				
	M12				
Cd + Hg	M16				
Cd + Pb +Hg	TP14				
	TP16				
	TP17				
	TP19				
	TP20				
	TP21				
	TP22				
	TP23				
	TP24				
	M9				
	M13				

## Supporting information

Table S5‐S11

## References

[fsn31636-bib-0001] Ajazuddin, S. S. (2012). Legal regulations of complementary and alternative medicines in different countries. Pharmacognosy Reviews 6(12), 154–160. 10.4103/0973-7847.99950 23055642 PMC3459458

[fsn31636-bib-0002] Avigan, M. I. , Mozersky, R. P. , & Seeff, L. B. (2016). Scientific and regulatory perspectives in herbal and dietary supplement associated hepatotoxicity in the united states. International Journal of Molecular Sciences, 17(3), 331 10.3390/ijms17030331 26950122 PMC4813193

[fsn31636-bib-0003] CEN, Comite Europeen de Normalisation . (2013). EN 13804:2013 Foodstuffs ‐ determination of elements and their chemical species ‐ general considerations and specific requirements.

[fsn31636-bib-0004] Chary, N. S. , Kamala, C. T. , & Raj, D. S. (2008). Assessing risk of heavy metals from consuming food grown on sewage irrigated soils and food chain transfer. Ecotoxicology and Environmental Safety, 69(3), 513–524. 10.1016/j.ecoenv.2007.04.013 17555815

[fsn31636-bib-0005] Czajkowska, D. (2006). Botanical and animal origin dietary supplements ‐ limits of chemical and microbiological contaminants. Food Industry, 7, 22–29. [in Polish].

[fsn31636-bib-0006] EC, European Commission . (2006). Commission Regulation (EC) No 1881/2006 of 19 December 2006 setting maximum levels for certain contaminants in foodstuffs (L 364, 20.12.2006).

[fsn31636-bib-0007] EC, European Commission . (2007). Commission Regulation (EC) No 333/2007 of 28 March 2007 laying down methods for the sampling and analysis for the control of levels of trace elements and process impurities in foodstuffs. (L 88, 29.3.2007).

[fsn31636-bib-0008] EC, European Commission . (2008). Commission Regulation (EC) No 629/2008 of 2 July 2008 amending Regulation (EC) No 1881/2006 setting maximum levels for certain contaminants in foodstuffs (L 173, 3.7.2008).

[fsn31636-bib-0009] EC, European Commission . (2011). Commission Regulation (EC) No 420/2011 of 29 April 2011 amending Regulation (EC) No 1881/2006 setting maximum levels for certain contaminants in foodstuffs (L, 111, 30.4.2011).

[fsn31636-bib-0010] EC, European Commission . (2014). Commission Regulation (EU) No 488/2014 of 12 May 2014 amending Regulation (EC) No 1881/2006 as regards maximum levels of cadmium in foodstuffs (L 138, 13.5.2014).

[fsn31636-bib-0011] EFSA, European Food Safety Authority . Food supplements. (2019). Introduction. https://www.efsa.europa.eu/en/topics/topic/food‐supplements. Accessed 15 May 2019.

[fsn31636-bib-0012] EP, European Parliament . (2002). Directive 2002/46/EC of the European Parliament and of the Council of 10 June 2002 on the approximation of the laws of the Member States relating to food supplements. (L, 183, 12.7.2002).

[fsn31636-bib-0013] Filipiak‐Szok, A. , Kurzawa, M. , & Szłyk, E. (2015). Determination of toxic metals by ICP‐MS in Asiatic and European medicinal plants and dietary supplements. Journal of Trace Elements in Medicine and Biology, 30, 54–58. 10.1016/j.jtemb.2014.10.008 25467854

[fsn31636-bib-0014] Flora, G. , Gupta, D. , & Tiwari, A. (2012). Toxicity of lead: A review with recent updates. Interdisciplinary Toxicology, 5(2), 47–58. 10.2478/v10102-012-0009-2 23118587 PMC3485653

[fsn31636-bib-0015] Gambuś, F. , & Rak, M. (2000). Influence of soil properties on the solubility of cadmium compounds. ZPPNR, 472(1), 251–257. [in Polish].

[fsn31636-bib-0016] IR, Industry Report. Dietary supplements market in Poland . (2017). Market analysis and development forecasts for 2017–2022. https://mypmr.pro/products/dietary‐supplements‐market‐in‐poland‐2017. Accessed 16 May 2019.

[fsn31636-bib-0017] Jaishankar, M. , Tseten, T. , Anbalagan, N. , Blessy, B. M. , & Krishnamurthy, N. B. (2014). Toxicity, mechanism and health effects of some heavy metals. Interdisciplinary Toxicology, 7(2), 60–72. 10.2478/intox-2014-0009 26109881 PMC4427717

[fsn31636-bib-0018] Jamal, Q. , Durani, P. , Khan, K. , Shahzad, M. , Munir, S. , Hussain, S. , … Anees, M. (2013). Heavy metals accumulation and their toxic effects: review. JBMS, 1(1–2), 27–36. http://citeseerx.ist.psu.edu/viewdoc/download?doi=10.1.1.1025.1708&rep=rep1&type=pdf. Accessed 16 May 2019.

[fsn31636-bib-0019] Jolly, Y. N. , Islam, A. , & Akbar, S. (2013). Transfer of metals from soil to vegetables and possible health risk assessment. Springer Plus, 2, 385 10.1186/2193-1801-2-385 24010043 PMC3755813

[fsn31636-bib-0020] Kaczyńska, A. , Zajączkowski, M. , & Grzybiak, M. (2015). Cadmium toxicity in plants and humans. Annales Academiae Medicae Gedanensis, 45, 65–70. [in Polish].

[fsn31636-bib-0021] Korfali, S. I. , Hawi, T. , & Mroueh, M. (2013). Evaluation of heavy metals content in dietary supplements in Lebanon. Chemistry Central Journal, 7, 10 10.1186/1752-153X-7-10 23331553 PMC3560192

[fsn31636-bib-0022] Kotynia, Z. , Szewczyk, P. , & Tuzikiewicz‐Gnitecka, G. (2017). Safety of dietary supplements ‐ admission to trading in Poland. State Control, 62(4), 49–61. [in Polish].

[fsn31636-bib-0023] Papanikolaou, N. C. , Hatzidaki, E. G. , Belivanis, S. , Tzanakakis, G. N. , & Tsatsakis, A. M. (2005). Lead toxicity update. A Brief Review. Medical Science Monitor, 11(10), 329–336. 16192916

[fsn31636-bib-0024] Rahimzadeh, R. M. , Rahimzadeh, R. M. , Kazemi, S. , & Moghadamnia, A. A. (2017). Cadmium toxicity and treatment: An update. Caspian Journal of Internal Medicine, 8(3), 135–145. 10.22088/cjim.8.3.135 28932363 PMC5596182

[fsn31636-bib-0025] Rattan, R. K. , Datta, S. P. , Chhonkar, P. K. , Suribabu, K. , & Singh, A. K. (2005). Long‐term impact of irrigation with sewage effluents on heavy metal content in soils, crops and groundwater – a case study. Agriculture, Ecosystems & Environment, 109, 310–322. 10.1016/j.agee.2005.02.025

[fsn31636-bib-0026] SAO, The Supreme Audit Office . (2016). Admission to the market of dietary supplements. LLO.430.002, Registration No 195/2016/P/16/078/LLO. https://www.nik.gov.pl/plik/id.13031.vp.15443.pdf. Accessed 09 February 2018.

[fsn31636-bib-0027] Schlegel‐Zawadzka, M. , & Barteczko, M. (2009). Evaluation of the natural supplements usage by adults for wholesome purposes. Food ‐ Science Technology Quality, 4(65), 375–387. [in Polish].

[fsn31636-bib-0028] Seńczuk, W. (2005). Modern toxicology. PZWL. Warszawa [in Polish]. ISBN: 978‐83‐200‐4506‐2.

[fsn31636-bib-0029] Socha, K. (2010). The estimation of content of lead in dietary supplements. Bromatologia I Chemia Toksykologiczna, 43(4), 529–532. [in Polish].

[fsn31636-bib-0030] Socha, K. , & Borawska, M. H. (2011). Evaluation of cadmium content in dietary supplements. Bromatologia I Chemia Toksykologiczna, 44(3), 351–354. [in Polish].

[fsn31636-bib-0031] Socha, K. , Michalska‐Mosiej, M. , Lipka‐Chudzik, K. , & Borawska, M. H. (2013). The mercury content in dietary supplements. Problemy Higieny I Epidemiologii, 94(3), 645–647. [in Polish].

[fsn31636-bib-0032] US EPA, United States Environmental Protection Agency . (1989). Risk Assessment Guidance for Superfund Volume I: Human Health Evaluation Manual (Part A). Washington, DC; EPA/540/1‐89/002.

[fsn31636-bib-0033] US EPA, United States Environmental Protection Agency . (2011). Exposure Factors Handbook 2011 Edition (Final Report). National Center for Environmental Assessment. Washington, DC; EPA/600/R‐09/052F.

