# Supplementary material for: Health risks from consumption of medicinal plant dietary supplements
Source: Food Sci Nutr. 2020 May 19;8(7):3535–44. doi: 10.1002/fsn3.1636 (PMC7382111; doi:10.1002/fsn3.1636)
Supplement: Supplementary file 1 — Table S5‐S11 [file FSN3-8-3535-s001.docx]

**Supplementary Material**

**Table 5. Average daily dietary exposure to Cd (mg kg^-1^ day^-1^) with dietary supplements and hazard quotient (HQ) depending on the dose**

| **Dietary supplement** | **Scenario I**  **MPC dose** | | **Scenario II**  **Daily dose** | | **Scenario III**  **Double-daily dose** | |
| --- | --- | --- | --- | --- | --- | --- |
|  | **ADD** | **HQ** | **ADD** | **HQ** | **ADD** | **HQ** |
| TP1 | 0.086 | 0.086 | 0.002 | 0.002 | 0.003 | 0.003 |
| TP4 | 0.086 | 0.086 | 0.006 | 0.006 | 0.013 | 0.013 |
| TP5 | 0.086 | 0.086 | 0.007 | 0.007 | 0.014 | 0.014 |
| TP8 | 0.043 | 0.043 | 0.001 | 0.001 | 0.003 | 0.003 |
| TP11 | 0.571 | 0.571 | 0.005 | 0.005 | 0.010 | 0.010 |
| TP14 | 0.057 | 0.057 | 0.007 | 0.007 | 0.015 | 0.015 |
| TP15 | 0.143 | 0.143 | 0.134 | 0.134 | 0.269 | 0.269 |
| TP16 | 0.143 | 0.143 | 0.001 | 0.001 | 0.001 | 0.001 |
| TP17 | 0.057 | 0.057 | 0.003 | 0.003 | 0.005 | 0.005 |
| TP18 | 0.110 | 0.110 | 0.001 | 0.001 | 0.002 | 0.002 |
| TP19 | 0.007 | 0.007 | 0.000 | 0.000 | 0.001 | 0.001 |
| TP20 | 0.097 | 0.097 | 0.019 | 0.019 | 0.039 | 0.039 |
| TP21 | 0.073 | 0.073 | 0.003 | 0.003 | 0.005 | 0.005 |
| TP22 | 0.171 | 0.171 | 0.017 | 0.017 | 0.034 | 0.034 |
| TP23 | 0.012 | 0.012 | 0.009 | 0.009 | 0.017 | 0.017 |
| TP24 | 0.073 | 0.073 | 0.006 | 0.006 | 0.011 | 0.011 |
| M2 | 0.149 | 0.050 | 0.003 | 0.001 | 0.006 | 0.002 |
| M3 | 0.165 | 0.055 | 0.001 | 0.000 | 0.001 | 0.000 |
| M5 | 0.165 | 0.055 | 0.003 | 0.001 | 0.006 | 0.002 |
| M6 | 0.064 | 0.021 | 0.002 | 0.001 | 0.003 | 0.001 |
| M7 | 0.129 | 0.043 | 0.003 | 0.001 | 0.005 | 0.002 |
| M8 | 0.198 | 0.066 | 0.001 | 0.000 | 0.002 | 0.001 |
| M9 | 0.129 | 0.043 | 0.001 | 0.000 | 0.002 | 0.001 |
| M10 | 0.129 | 0.043 | 0.001 | 0.000 | 0.002 | 0.001 |
| M11 | 0.055 | 0.018 | 0.000 | 0.000 | 0.001 | 0.000 |
| M12 | 0.429 | 0.143 | 0.006 | 0.002 | 0.012 | 0.004 |
| M13 | 0.055 | 0.018 | 0.007 | 0.002 | 0.014 | 0.005 |
| M16 | 0.214 | 0.071 | 0.003 | 0.001 | 0.005 | 0.002 |

**Table 6. Average daily dietary exposure to Cd (mg kg^-1^ day^-1^) with dietary supplements and hazard quotient (HQ) depending on the period of intake**

| **Dietary supplement** | **Scenario IV**  **2 weeks intake** | | **Scenario V**  **1 month intake** | | **Scenario VI**  **2 month intake** | | **Scenario VII**  **3 month intake** | |
| --- | --- | --- | --- | --- | --- | --- | --- | --- |
|  | **ADD** | **HQ** | **ADD** | **HQ** | **ADD** | **HQ** | **ADD** | **HQ** |
| TP1 | 0.024 | 0.024 | 0.048 | 0.048 | 0.096 | 0.096 | 0.144 | 0.144 |
| TP4 | 0.090 | 0.090 | 0.180 | 0.180 | 0.360 | 0.360 | 0.540 | 0.540 |
| TP5 | 0.096 | 0.096 | 0.192 | 0.192 | 0.384 | 0.384 | 0.576 | 0.576 |
| TP8 | 0.020 | 0.020 | 0.040 | 0.040 | 0.079 | 0.079 | 0.119 | 0.119 |
| TP11 | 0.072 | 0.072 | 0.144 | 0.144 | 0.288 | 0.288 | 0.432 | 0.432 |
| TP14 | 0.104 | 0.104 | 0.208 | 0.208 | 0.416 | 0.416 | 0.624 | 0.624 |
| TP15 | 1.880 | **1.880** | 3.760 | **3.760** | 7.520 | **7.520** | 11.280 | **11.280** |
| TP16 | 0.008 | 0.008 | 0.016 | 0.016 | 0.032 | 0.032 | 0.048 | 0.048 |
| TP17 | 0.036 | 0.036 | 0.072 | 0.072 | 0.144 | 0.144 | 0.216 | 0.216 |
| TP18 | 0.017 | 0.017 | 0.034 | 0.034 | 0.068 | 0.068 | 0.101 | 0.101 |
| TP19 | 0.005 | 0.005 | 0.009 | 0.009 | 0.019 | 0.019 | 0.028 | 0.028 |
| TP20 | 0.272 | 0.272 | 0.544 | 0.544 | 1.088 | **1.088** | 1.632 | **1.632** |
| TP21 | 0.037 | 0.037 | 0.073 | 0.073 | 0.147 | 0.147 | 0.220 | 0.220 |
| TP22 | 0.238 | 0.238 | 0.475 | 0.475 | 0.950 | 0.950 | 1.426 | **1.426** |
| TP23 | 0.121 | 0.121 | 0.243 | 0.243 | 0.486 | 0.486 | 0.728 | 0.728 |
| TP24 | 0.078 | 0.078 | 0.155 | 0.155 | 0.310 | 0.310 | 0.465 | 0.465 |
| M2 | 0.043 | 0.014 | 0.086 | 0.029 | 0.172 | 0.057 | 0.258 | 0.086 |
| M3 | 0.009 | 0.003 | 0.018 | 0.006 | 0.037 | 0.012 | 0.055 | 0.018 |
| M5 | 0.043 | 0.014 | 0.086 | 0.029 | 0.172 | 0.057 | 0.258 | 0.086 |
| M6 | 0.023 | 0.008 | 0.047 | 0.016 | 0.094 | 0.031 | 0.140 | 0.047 |
| M7 | 0.038 | 0.013 | 0.076 | 0.025 | 0.151 | 0.050 | 0.227 | 0.076 |
| M8 | 0.011 | 0.004 | 0.022 | 0.007 | 0.044 | 0.015 | 0.067 | 0.022 |
| M9 | 0.014 | 0.005 | 0.029 | 0.010 | 0.058 | 0.019 | 0.086 | 0.029 |
| M10 | 0.013 | 0.004 | 0.026 | 0.009 | 0.053 | 0.018 | 0.079 | 0.026 |
| M11 | 0.005 | 0.002 | 0.010 | 0.003 | 0.019 | 0.006 | 0.029 | 0.010 |
| M12 | 0.082 | 0.027 | 0.164 | 0.055 | 0.328 | 0.109 | 0.492 | 0.164 |
| M13 | 0.095 | 0.032 | 0.190 | 0.063 | 0.381 | 0.127 | 0.571 | 0.190 |
| M16 | 0.036 | 0.012 | 0.072 | 0.024 | 0.144 | 0.048 | 0.216 | 0.072 |

**Table 7. Average daily dietary exposure to Pb (mg kg^-1^ day^-1^) with dietary supplements and hazard quotient (HQ) depending on the dose**

| **Dietary supplement** | **Scenario I**  **MPC dose** | | **Scenario II**  **Daily dose** | | **Scenario III**  **Double-daily dose** | |
| --- | --- | --- | --- | --- | --- | --- |
|  | **ADD** | **HQ** | **ADD** | **HQ** | **ADD** | **HQ** |
| TP5 | 0.257 | 0.086 | 0.016 | 0.005 | 0.033 | 0.011 |
| TP6 | 0.064 | 0.021 | 0.002 | 0.001 | 0.005 | 0.002 |
| TP7 | 0.257 | 0.086 | 0.009 | 0.003 | 0.017 | 0.006 |
| TP12 | 0.857 | 0.286 | 0.006 | 0.002 | 0.012 | 0.004 |
| TP14 | 0.171 | 0.057 | 0.026 | 0.009 | 0.053 | 0.018 |
| TP15 | 0.429 | 0.143 | 0.030 | 0.010 | 0.060 | 0.020 |
| TP16 | 0.429 | 0.143 | 0.016 | 0.005 | 0.031 | 0.010 |
| TP17 | 0.171 | 0.057 | 0.008 | 0.003 | 0.016 | 0.005 |
| TP18 | 0.329 | 0.110 | 0.005 | 0.002 | 0.010 | 0.003 |
| TP19 | 0.021 | 0.007 | 0.007 | 0.002 | 0.015 | 0.005 |
| TP20 | 0.291 | 0.097 | 0.449 | 0.150 | 0.899 | 0.300 |
| TP21 | 0.219 | 0.073 | 0.021 | 0.007 | 0.042 | 0.014 |
| TP22 | 0.514 | 0.171 | 0.189 | 0.063 | 0.377 | 0.126 |
| TP23 | 0.036 | 0.012 | 0.133 | 0.044 | 0.265 | 0.088 |
| TP24 | 0.219 | 0.073 | 0.054 | 0.018 | 0.108 | 0.036 |
| M3 | 0.165 | 0.055 | 0.017 | 0.006 | 0.035 | 0.012 |
| M4 | 0.085 | 0.028 | 0.019 | 0.006 | 0.038 | 0.013 |
| M5 | 0.165 | 0.055 | 0.013 | 0.004 | 0.026 | 0.009 |
| M6 | 0.064 | 0.021 | 0.006 | 0.002 | 0.012 | 0.004 |
| M7 | 0.129 | 0.043 | 0.003 | 0.001 | 0.005 | 0.002 |
| M8 | 0.198 | 0.066 | 0.012 | 0.004 | 0.024 | 0.008 |
| M9 | 0.129 | 0.043 | 0.003 | 0.001 | 0.007 | 0.002 |
| M10 | 0.129 | 0.043 | 0.003 | 0.001 | 0.007 | 0.002 |
| M11 | 0.055 | 0.018 | 0.001 | 0.000 | 0.002 | 0.001 |
| M12 | 0.429 | 0.143 | 0.010 | 0.003 | 0.019 | 0.006 |
| M13 | 0.055 | 0.018 | 0.042 | 0.014 | 0.083 | 0.028 |
| M14 | 0.249 | 0.083 | 0.011 | 0.004 | 0.022 | 0.007 |
| M15 | 0.249 | 0.083 | 0.006 | 0.002 | 0.013 | 0.004 |

**Table 8. Average daily dietary exposure to Pb (mg kg^-1^ day^-1^) with dietary supplements and hazard quotient (HQ) depending on the period of intake**

| **Dietary supplement** | **Scenario IV**  **2 weeks intake** | | **Scenario V**  **1 month intake** | | **Scenario VI**  **2 month intake** | | **Scenario VII**  **3 month intake** | |
| --- | --- | --- | --- | --- | --- | --- | --- | --- |
|  | **ADD** | **HQ** | **ADD** | **HQ** | **ADD** | **HQ** | **ADD** | **HQ** |
| TP5 | 0.229 | 0.076 | 0.458 | 0.153 | 0.917 | 0.306 | 1.375 | 0.458 |
| TP6 | 0.032 | 0.011 | 0.065 | 0.022 | 0.130 | 0.043 | 0.194 | 0.065 |
| TP7 | 0.121 | 0.040 | 0.241 | 0.080 | 0.482 | 0.161 | 0.724 | 0.241 |
| TP12 | 0.084 | 0.028 | 0.168 | 0.056 | 0.336 | 0.112 | 0.504 | 0.168 |
| TP14 | 0.368 | 0.123 | 0.736 | 0.245 | 1.472 | 0.491 | 2.208 | 0.736 |
| TP15 | 0.420 | 0.140 | 0.840 | 0.280 | 0.448 | 0.149 | 0.672 | 0.224 |
| TP16 | 0.220 | 0.073 | 0.440 | 0.147 | 0.289 | 0.096 | 0.433 | 0.144 |
| TP17 | 0.112 | 0.037 | 0.224 | 0.075 | 1.680 | 0.560 | 2.520 | 0.840 |
| TP18 | 0.072 | 0.024 | 0.144 | 0.048 | 0.880 | 0.293 | 1.320 | 0.440 |
| TP19 | 0.104 | 0.035 | 0.207 | 0.069 | 0.414 | 0.138 | 0.621 | 0.207 |
| TP20 | 6.290 | **2.097** | 12.580 | **4.193** | 25.160 | **8.387** | 37.740 | **12.580** |
| TP21 | 0.296 | 0.099 | 0.592 | 0.197 | 1.183 | 0.394 | 1.775 | 0.592 |
| TP22 | 2.640 | 0.880 | 5.280 | **1.760** | 10.560 | **3.520** | 15.840 | **5.280** |
| TP23 | 1.856 | 0.619 | 3.713 | **1.238** | 7.426 | **2.475** | 11.138 | **3.713** |
| TP24 | 0.755 | 0.252 | 1.510 | 0.503 | 3.019 | **1.006** | 4.529 | **1.510** |
| M3 | 0.243 | 0.081 | 0.485 | 0.162 | 0.619 | 0.206 | 0.929 | 0.310 |
| M4 | 0.269 | 0.090 | 0.539 | 0.180 | 0.971 | 0.324 | 1.456 | 0.485 |
| M5 | 0.184 | 0.061 | 0.369 | 0.123 | 1.077 | 0.359 | 1.616 | 0.539 |
| M6 | 0.081 | 0.027 | 0.162 | 0.054 | 0.362 | 0.121 | 0.543 | 0.181 |
| M7 | 0.038 | 0.013 | 0.077 | 0.026 | 0.737 | 0.246 | 1.106 | 0.369 |
| M8 | 0.166 | 0.055 | 0.333 | 0.111 | 0.324 | 0.108 | 0.486 | 0.162 |
| M9 | 0.047 | 0.016 | 0.095 | 0.032 | 0.154 | 0.051 | 0.230 | 0.077 |
| M10 | 0.046 | 0.015 | 0.091 | 0.030 | 0.665 | 0.222 | 0.998 | 0.333 |
| M11 | 0.017 | 0.006 | 0.034 | 0.011 | 2.335 | 0.778 | 3.502 | **1.167** |
| M12 | 0.134 | 0.045 | 0.268 | 0.089 | 0.190 | 0.063 | 0.284 | 0.095 |
| M13 | 0.584 | 0.195 | 1.167 | 0.389 | 0.182 | 0.061 | 0.274 | 0.091 |
| M14 | 0.155 | 0.052 | 0.310 | 0.103 | 0.068 | 0.023 | 0.101 | 0.034 |
| M15 | 0.090 | 0.030 | 0.181 | 0.060 | 0.536 | 0.179 | 0.804 | 0.268 |

**Table 9. Average daily dietary exposure to Hg (mg kg^-1^ day^-1^) with dietary supplements and hazard quotient (HQ) depending of the dose**

| **Dietary supplement** | **Scenario I**  **MPC dose** | | **Scenario II**  **Daily dose** | | **Scenario III**  **Double-daily dose** | |
| --- | --- | --- | --- | --- | --- | --- |
|  | **ADD** | **HQ** | **ADD** | **HQ** | **ADD** | **HQ** |
| TP14 | 0.006 | 0.057 | 0.001 | 0.007 | 0.001 | 0.015 |
| TP16 | 0.014 | 0.143 | 0.001 | 0.013 | 0.003 | 0.025 |
| TP17 | 0.006 | 0.057 | 0.000 | 0.005 | 0.001 | 0.009 |
| TP19 | 0.001 | 0.007 | 0.000 | 0.001 | 0.000 | 0.001 |
| TP20 | 0.010 | 0.097 | 0.003 | 0.034 | 0.007 | 0.068 |
| TP21 | 0.007 | 0.073 | 0.000 | 0.004 | 0.001 | 0.009 |
| TP22 | 0.017 | 0.171 | 0.004 | 0.043 | 0.009 | 0.086 |
| TP23 | 0.001 | 0.012 | 0.001 | 0.007 | 0.001 | 0.013 |
| TP24 | 0.007 | 0.073 | 0.001 | 0.012 | 0.002 | 0.023 |
| M9 | 0.004 | 0.043 | 0.000 | 0.003 | 0.001 | 0.007 |
| M13 | 0.002 | 0.018 | 0.002 | 0.018 | 0.004 | 0.035 |
| M16 | 0.007 | 0.071 | 0.001 | 0.012 | 0.002 | 0.024 |

**Table 10. Average daily dietary exposure to Hg (mg kg^-1^ day^-1^) with dietary supplements and hazard quotient (HQ) depending on the period of intake**

| **Dietary supplement** | **Scenario IV**  **2 weeks intake** | | **Scenario V**  **1 month intake** | | **Scenario VI**  **2 month intake** | | **Scenario VII**  **3 month intake** | |
| --- | --- | --- | --- | --- | --- | --- | --- | --- |
|  | **ADD** | **HQ** | **ADD** | **HQ** | **ADD** | **HQ** | **ADD** | **HQ** |
| TP14 | 0.010 | 0.104 | 0.021 | 0.208 | 0.042 | 0.416 | 0.062 | 0.624 |
| TP16 | 0.018 | 0.178 | 0.036 | 0.356 | 0.026 | 0.256 | 0.038 | 0.384 |
| TP17 | 0.006 | 0.064 | 0.013 | 0.128 | 0.071 | 0.712 | 0.107 | **1.068** |
| TP19 | 0.001 | 0.009 | 0.002 | 0.018 | 0.004 | 0.036 | 0.005 | 0.054 |
| TP20 | 0.048 | 0.476 | 0.095 | 0.952 | 0.190 | **1.904** | 0.286 | **2.856** |
| TP21 | 0.006 | 0.061 | 0.012 | 0.122 | 0.024 | 0.245 | 0.037 | 0.367 |
| TP22 | 0.060 | 0.600 | 0.120 | **1.200** | 0.240 | **2.400** | 0.360 | **3.600** |
| TP23 | 0.009 | 0.093 | 0.019 | 0.186 | 0.037 | 0.371 | 0.056 | 0.557 |
| TP24 | 0.016 | 0.163 | 0.033 | 0.326 | 0.065 | 0.653 | 0.098 | 0.979 |
| M9 | 0.005 | 0.048 | 0.010 | 0.096 | 0.068 | 0.680 | 0.102 | **1.020** |
| M13 | 0.025 | 0.246 | 0.049 | 0.492 | 0.098 | 0.983 | 0.147 | **1.475** |
| M16 | 0.017 | 0.170 | 0.034 | 0.340 | 0.019 | 0.192 | 0.029 | 0.288 |

**Table 11. Hazard index (HI) for chronic exposure to heavy metals depending on the period of dietary supplements intake**

| **Cumulative effect of heavy metals exposure** | **Dietary supplement** | **Scenario I** | **Scenario II** | **Scenario III** | **Scenario IV** | **Scenario V** | **Scenario VI** | **Scenario VII** |
| --- | --- | --- | --- | --- | --- | --- | --- | --- |
|  |  | **MPC dose** | **Daily dose** | **Double-daily dose** | **2 weeks intake** | **1 month intake** | **2 month intake** | **3 month intake** |
| **Cd + Pb** | TP5 | 0.172 | 0.012 | 0.025 | 0.172 | 0.345 | 0.690 | **1.034** |
|  | TP15 | 0.286 | 0.144 | 0.289 | **2.020** | **4.040** | **7.669** | **11.504** |
|  | TP18 | 0.220 | 0.003 | 0.005 | 0.041 | 0.082 | 0.361 | 0.541 |
|  | M3 | 0.110 | 0.006 | 0.012 | 0.084 | 0.168 | 0.218 | 0.328 |
|  | M5 | 0.110 | 0.005 | 0.011 | 0.075 | 0.152 | 0.416 | 0.625 |
|  | M6 | 0.042 | 0.003 | 0.005 | 0.035 | 0.070 | 0.152 | 0.228 |
|  | M7 | 0.086 | 0.002 | 0.004 | 0.026 | 0.051 | 0.296 | 0.445 |
|  | M8 | 0.132 | 0.004 | 0.009 | 0.059 | 0.118 | 0.123 | 0.184 |
|  | M10 | 0.086 | 0.001 | 0.003 | 0.019 | 0.039 | 0.240 | 0.359 |
|  | M11 | 0.036 | 0.000 | 0.001 | 0.008 | 0.014 | 0.784 | **1.177** |
|  | M12 | 0.286 | 0.005 | 0.010 | 0.072 | 0.144 | 0.172 | 0.259 |
| **Cd + Hg** | M16 | 0.142 | 0.013 | 0.026 | 0.182 | 0.364 | 0.240 | 0.360 |
| **Cd + Pb + Hg** | TP14 | 0.171 | 0.023 | 0.048 | 0.331 | 0.661 | **1.323** | **1.984** |
|  | TP16 | 0.429 | 0.019 | 0.036 | 0.259 | 0.519 | 0.384 | 0.576 |
|  | TP17 | 0.171 | 0.011 | 0.019 | 0.137 | 0.275 | **1.416** | **2.124** |
|  | TP19 | 0.021 | 0.003 | 0.007 | 0.049 | 0.096 | 0.193 | 0.289 |
|  | TP20 | 0.291 | 0.203 | 0.407 | **2.845** | **5.689** | **11.379** | **17.068** |
|  | TP21 | 0.219 | 0.014 | 0.028 | 0.197 | 0.392 | 0.786 | **1.179** |
|  | TP22 | 0.513 | 0.123 | 0.246 | **1.718** | **3.435** | **6.870** | **10.306** |
|  | TP23 | 0.036 | 0.060 | 0.118 | 0.833 | **1.667** | **3.332** | **4.998** |
|  | TP24 | 0.219 | 0.036 | 0.070 | 0.493 | 0.984 | **1.969** | **2.954** |
|  | M9 | 0.129 | 0.004 | 0.010 | 0.069 | 0.138 | 0.750 | **1.126** |
|  | M13 | 0.054 | 0.034 | 0.068 | 0.473 | 0.944 | **1.171** | **1.756** |
